# Supplementary figures and images for: Serotype skewing and immune imprinting shape response to the tetravalent dengue virus Qdenga vaccine
Source: medRxiv. 2026 Jun 26:2026.06.15.26355542. Preprint. [Version 1] doi: 10.64898/2026.06.15.26355542 (PMC13321214; doi:10.64898/2026.06.15.26355542)

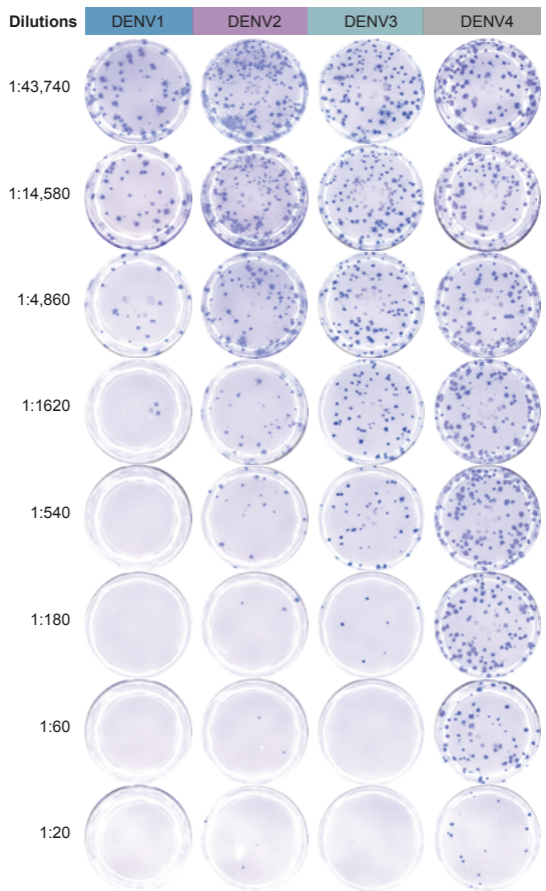

Suppl. Fig. 1

Supplement: Supplement 1 — Supplementary Data Figure 1: Representative FRNT neutralization curves. Representative plaque morphology for each DENV serotype used in the focus reduction neutralization assay (FRNT). Titration curves from a representative DENV-seropositive vaccinated individual at 60 days post-second Qdenga dose. Neutralization curves are shown for all four vaccine DENV serotypes (DENV-1–4). Participants were stratified by baseline serostatus: DENV-naïve (blue, n = 50) and DENV-exposed (purple, n = 49). Plasma was serially diluted in 8 threefold dilution steps, ranging from 1:20 to 1:43,720. [file media-1.pdf]

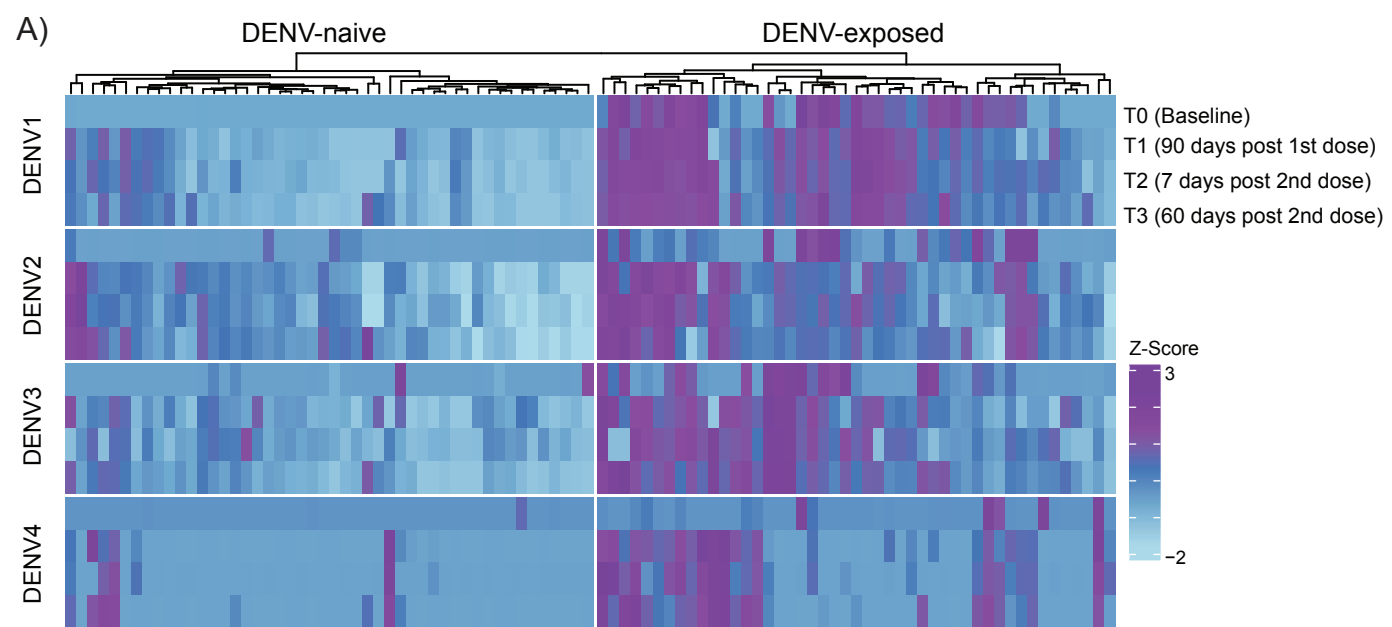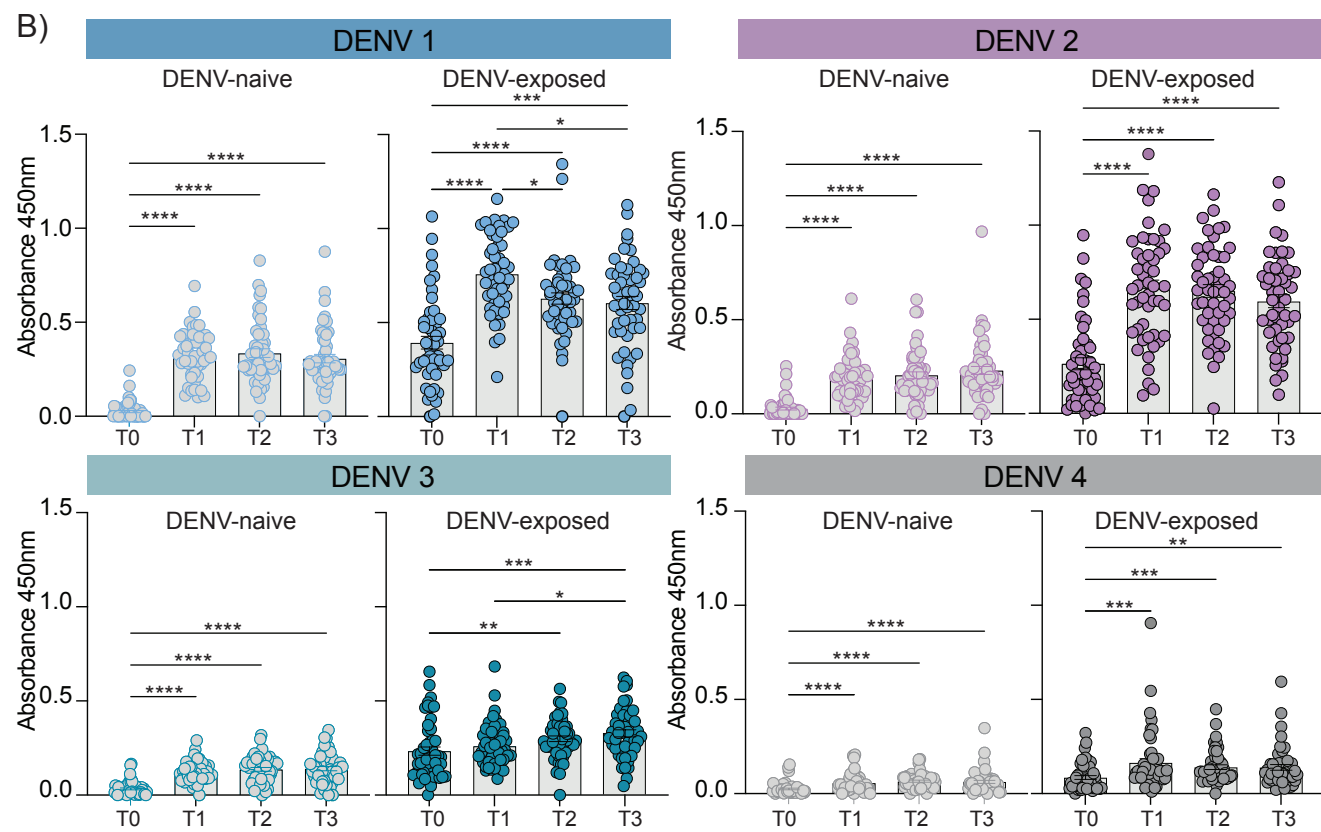

Supplement: Supplement 4 — Supplementary Data Figure 4. Neutralization profile and antibody binding induced by Qdenga vaccination. Plasma samples were collected at four time points: baseline (T0, prior to first dose), 90 days post-first dose/before the second dose (T1), 7 days post-second dose (T2), and 60 days post-second dose (T3). a, Heatmap of neutralization profiles (FRNT50) against vaccine DENV strains across all four timepoints (T0–T3), separated by baseline DENV serostatus: DENV-naive (left) and DENV-exposed (right) participants. Columns represent patient-specific FRNT50 responses, z-score normalized per serotype and timepoint. b, Plasma IgG binding reactivity against DENV-1–4 was assessed by whole-virus ELISA (1:600 serum dilution, OD 450 nm) using vaccine strains at all time points. Each plate was coated with 1 × 10^5^ PFU of the corresponding virus. [file media-4.pdf]

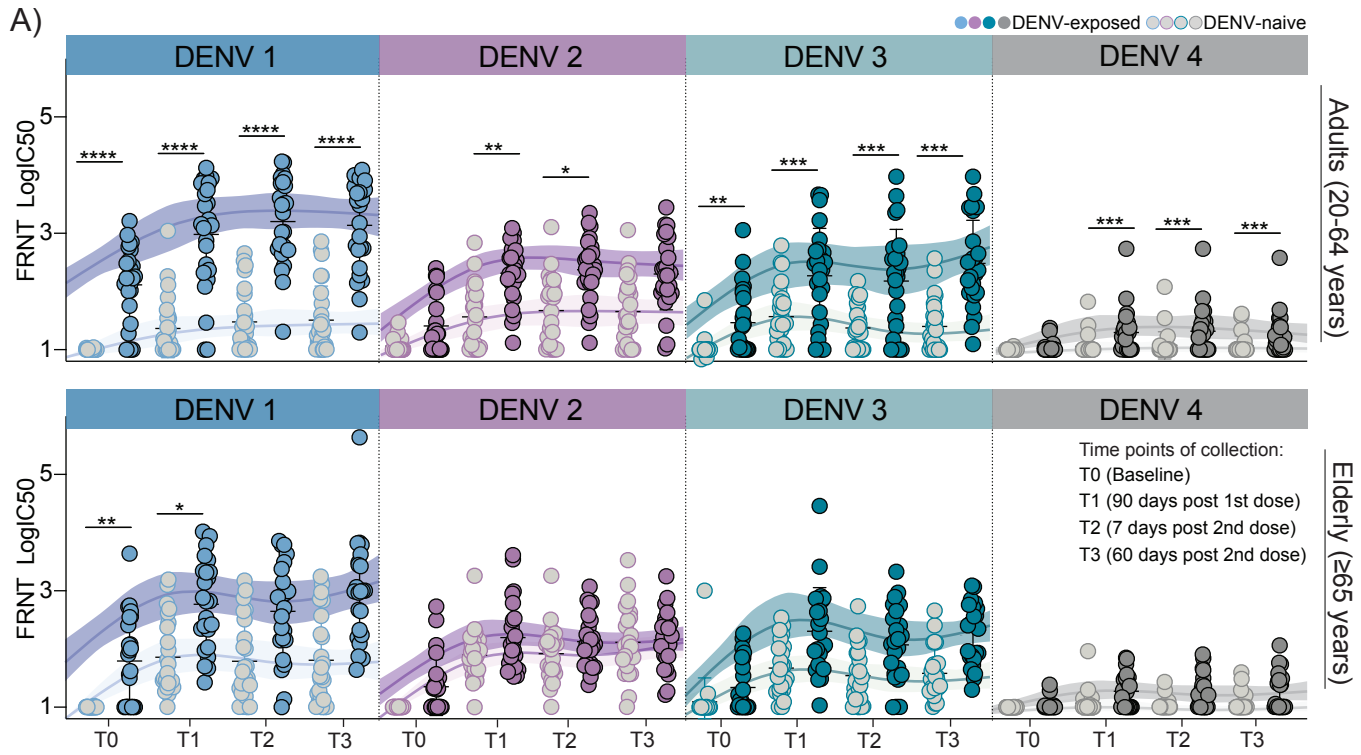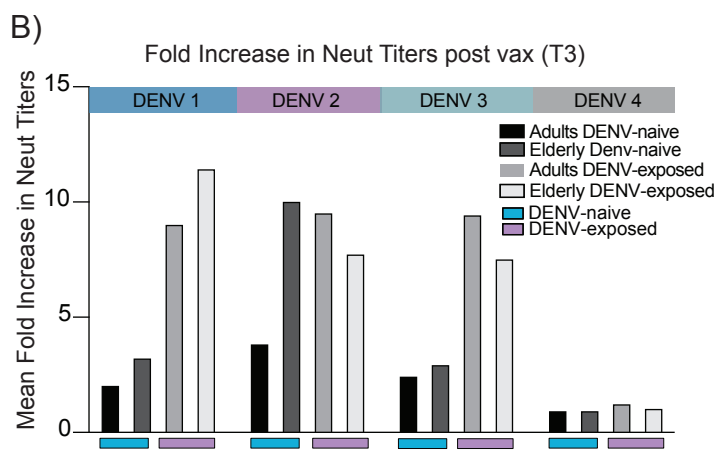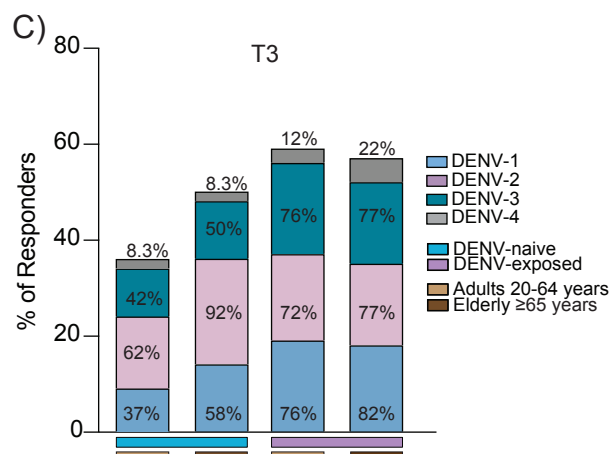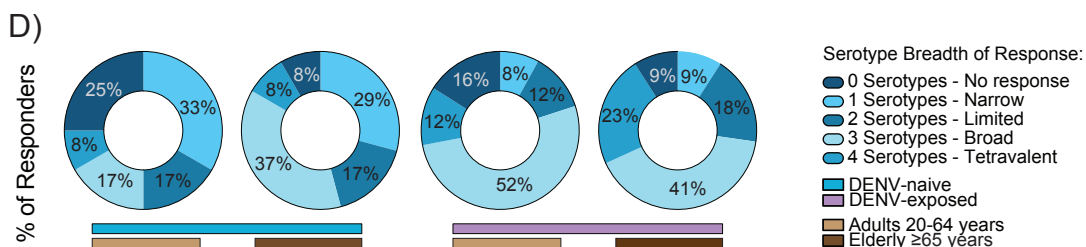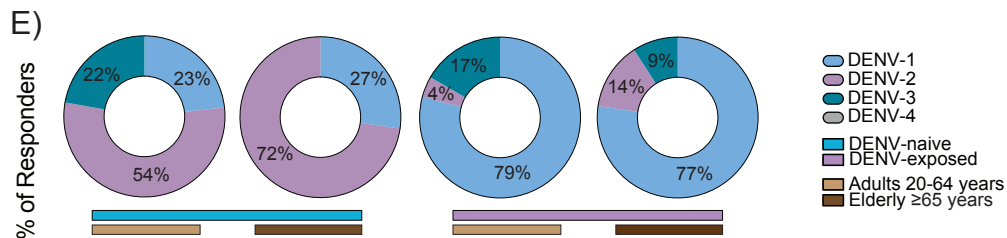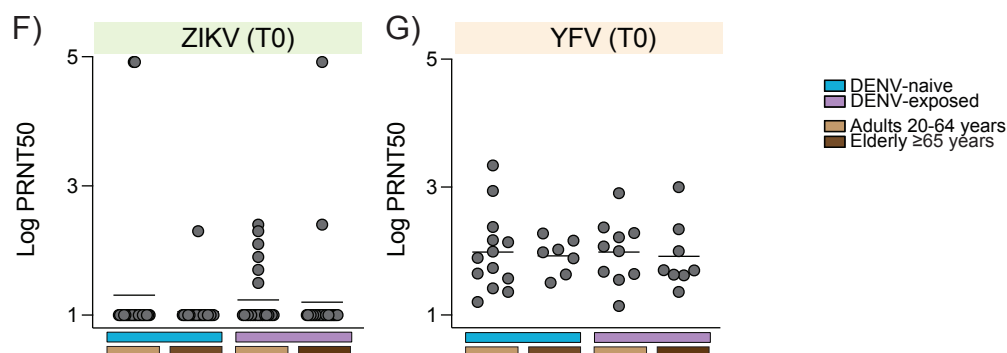

Supplement: Supplement 6 — Supplementary Data Figure 8. Impact of age on Qdenga immunogenicity. Plasma samples were collected at baseline (T0, prior to first dose), 90 days post-first dose/before the second dose (T1), 7 days post-second dose (T2), and 60 days post-second dose (T3). Participants received two doses of Qdenga at a 3-month interval and were stratified by age group, adults (20–64 years) and elderly (≥65 years), and by DENV baseline serostatus. a, Neutralization antibody levels against the four vaccine DENV serotypes (DENV-1 to DENV-4) were quantified across all time points using a focus reduction neutralization test (FRNT). b, Mean fold increase in neutralization titers at T3 for DENV-1 to DENV-4, stratified by age and serostatus: DENV-naïve adults (black), DENV-exposed adults (gray), DENV-naïve elderly (dark gray), and DENV-exposed elderly (light gray). c, Stacked bar plots representing the percentage of vaccine responders for each serotype at T3. Responders were defined as individuals who achieved a ≥0.5 log (3-fold) increase in neutralization titers relative to T0. Data shown for DENV-1 (blue), DENV-2 (purple), DENV-3 (green), and DENV-4 (gray). Age groups are denoted in light brown (adults) and dark brown (elderly). d, Pie chart distribution of immunization breadth after full vaccination, stratified by age and baseline serostatus. Individuals were categorized based on the number of DENV serotypes eliciting a neutralizing response: 0 (no response), 1 (narrow), 2 (limited), 3 (broad), or 4 (tetravalent). e, Percentage of individuals exhibiting serotype dominance at T3, defined as the serotype with the highest neutralization titer, stratified by age and serostatus. f, Neutralization titers against Zika virus at T0, stratified by age and DENV serostatus (n = 97 participants). g, Neutralization titers against yellow fever virus strain 17D at T0 in individuals with confirmed yellow fever vaccination, stratified by age and DENV serostatus (n = 38 participants). ZIKV, Zika virus. YFV, [file media-8.pdf]

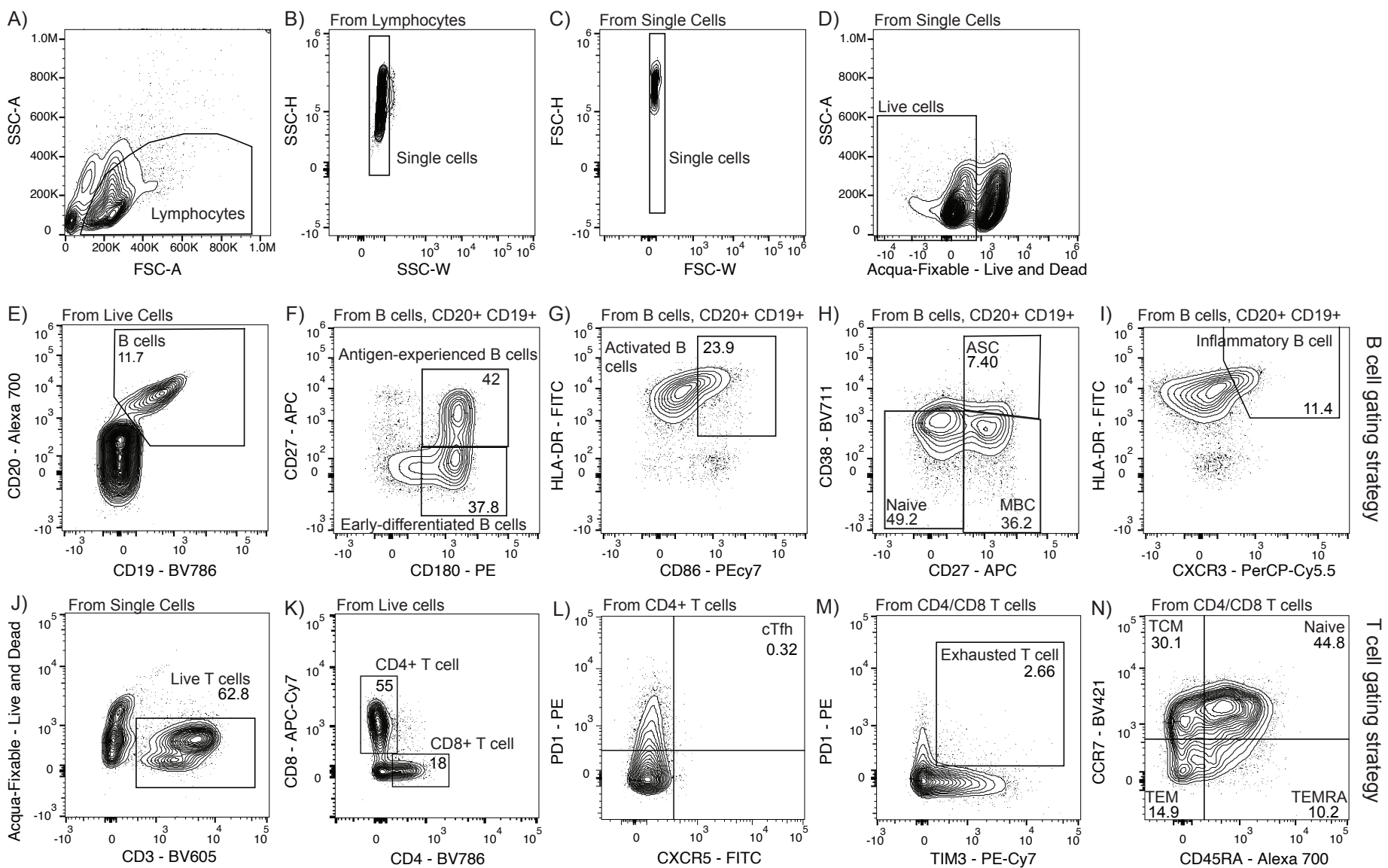

Supplement: Supplement 7 — Supplementary Data Fig. 9. Gating strategies for B and T cell subpopulations. a–d, Representative flow-cytometry gating strategy used to define major T- and B-cell subsets from peripheral blood mononuclear cells (PBMCs). Lymphocytes were first identified based on (a) forward- and side-scatter parameters (FSC-A/SSC-A), (b) followed by exclusion of doublets using SSC-H vs SSC-W and (c) FSC-H vs FSC-W, and (d) selection of live cells using a viability dye. e–i, B cell populations. Total B cells were defined as CD19+CD20+ (e). Within this population, the following subpopulations were identified: (f, bottom right) early-differentiated B cells (CD19+CD20+CD27−CD180+); (f, top right) antigen-experienced B cells (CD19+CD20+CD27+CD180+); (g) activated B cells (CD19+CD20+HLA-DR+CD86+); (h, bottom left) naïve B cells (CD19+CD20+CD27−CD38−); (h, bottom right) memory B cells (MBC; CD19+CD20+CD27+CD38−); (h, top right) antibody-secreting cells (ASC; CD19+CD20+CD27+CD38+); and (i) inflammatory B cells (CD19+CD20+HLA-DR+CXCR3+). j–q, T cell populations. (j) T cells were first gated on live CD3+ single cells (viability dye-negative; CD3+Aqua−). CD4+ and CD8+ subsets were then defined (k), and within each, functional populations were delineated as follows: l, circulating T follicular helper cells (cTfh; CD4+PD-1+CXCR5+); m, exhausted T cells (TIM-3+PD-1+); n, naïve T cells (CCR7+CD45RA+), central memory (TCM; CCR7+CD45RA−), effector memory (TEM; CCR7−CD45RA−), and terminally differentiated effector memory cells (TEMRA; CCR7−CD45RA+). In each plot, each dot represents a single cell, and the gated populations of interest are outlined by rectangular regions. The percentage displayed within each gate represents the frequency of that subset relative to its parent population. [file media-9.pdf]
