# Supplementary material for: Serotype skewing and immune imprinting shape response to the tetravalent dengue virus Qdenga vaccine
Source: medRxiv. 2026 Jun 26:2026.06.15.26355542. Preprint. [Version 1] doi: 10.64898/2026.06.15.26355542 (PMC13321214; doi:10.64898/2026.06.15.26355542)
Supplement: Supplement 5 — Supplementary Data Figure 7. Breadth and serotype dominance of vaccinated cohort. a, Pie chart depicting baseline serostatus distribution among individuals achieving the immunization threshold for ≥3 serotypes. DENV-exposed (blue) and DENV-naïve (white). b, Treemap representation of dominant serotype response among individuals achieving the immunization threshold for only one serotype, stratified by baseline serostatus. c, Treemap representation of dominant serotype response among individuals achieving the immunization threshold for only one serotype, stratified by baseline serostatus. DENV-exposed (left, dark) and DENV-naïve (right, light). Responders were defined as individuals achieving a ≥0.5 log (3-fold) increase in neutralization titers relative to their own baseline (T0). Only serotypes for which participants met the defined immunization threshold (≥3-fold increase from baseline) are represented d, Pie chart depicting baseline DENV serotype distribution among seropositive individuals (DENV-exposed). e, Alluvial plots showing the distribution of DENV-reactive CD4+ and CD8+ AIM+ T-cell responses across participants stratified by antibody-response breadth (no response, narrow (1 serotype), limited (2 serotypes), broad (3 serotypes), and tetravalent (4 serotypes). The width of each flow indicates the relative frequency of participants within each category, shown as percentages. T-cell responses were measured 60 days after the second vaccine dose and compared with corresponding antibody-response profiles (n = 79). Positive T-cell responses were defined as AIM+ frequencies above background-subtracted unstimulated controls and exceeding the predefined positivity threshold described in the Methods section. f, Stacked bar plots showing demographic characteristics (DENV baseline serostatus, sex, age group, hierarchical cluster assignment, yellow fever vaccination history, ZIKV baseline serostatus, breadth of response, and serotype dominance), of top responders. Best re [file media-7.pdf]

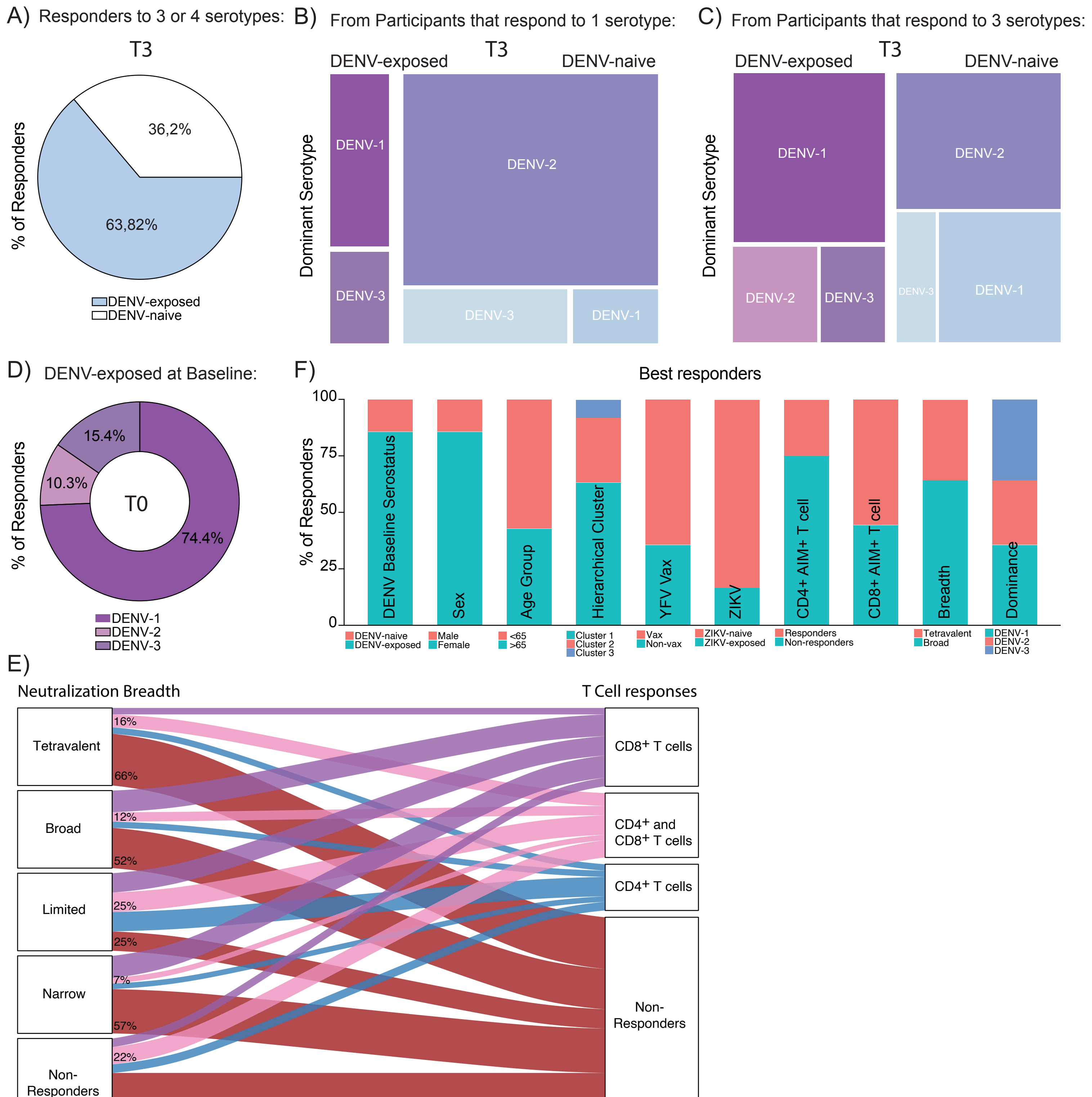
A) Responders to 3 or 4 serotypes:
T3
% of Responders
36,2%
63,82%
DENV-exposed
DENV-naive
B) From Participants that respond to 1 serotype:
T3
DENV-exposed
DENV-naive
Dominant Serotype
DENV-1
DENV-3
DENV-2
DENV-3
DENV-1
C) From Participants that respond to 3 serotypes:
T3
DENV-exposed
DENV-naive
Dominant Serotype
DENV-1
DENV-2
DENV-2
DENV-3
DENV-3
DENV-1
D) DENV-exposed at Baseline:
% of Responders
T0
15.4%
10.3%
74.4%
DENV-1
DENV-2
DENV-3
F) Best responders
% of Responders
100
75
50
25
0
DENV Baseline Serostatus
Sex
Age Group
Hierarchical Cluster
YFV Vax
ZIKV
CD4+ AIM+ T cell
CD8+ AIM+ T cell
Breadth
Dominance
DENV-naive
DENV-exposed
Male
Female
<65
>65
Cluster 1
Cluster 2
Cluster 3
Vax
Non-vax
ZIKV-naive
ZIKV-exposed
Responders
Non-responders
Tetravalent
Broad
DENV-1
DENV-2
DENV-3
E)
Neutralization Breadth
T Cell responses
Tetravalent
16%
66%
Broad
12%
52%
Limited
25%
25%
Narrow
7%
57%
Non-Responders
22%
55%
CD8+ T cells
CD4+ and CD8+ T cells
CD4+ T cells
Non-Responders

Suppl. Fig. 7
