# Supplementary material for: Serotype skewing and immune imprinting shape response to the tetravalent dengue virus Qdenga vaccine
Source: medRxiv. 2026 Jun 26:2026.06.15.26355542. Preprint. [Version 1] doi: 10.64898/2026.06.15.26355542 (PMC13321214; doi:10.64898/2026.06.15.26355542)
Supplement: Supplement 8 — Table S1. Cohort Demographics. Clinical and demographic data for the participants enrolled in the Qdenga Study. Information is presented for the overall cohort and stratified by baseline DENV serostatus into DENV-naïve and DENV-exposed groups. For each category, total numbers are provided alongside the corresponding percentage of participants (shown in parentheses). Immunization breadth was defined based on the number of DENV serotypes eliciting a detectable neutralizing antibody response post-vaccination: 0 = no response; 1 = narrow response; 2 = limited response; 3 = broad response; 4 = tetravalent response. [file media-10.pdf]

	Overall (N = 99)	DENV-naive (N = 50)	DENV-exposed (N = 49)
**Age Category**			
18 - 64 years	51 (51.5)	26 (52)	25 (51)
65 - 99 years	48 (48.5)	24 (48)	24 (49)
**Sex**			
Female	69 (69.7)	35 (70)	34 (69.4)
Male	30 (30.3)	15 (30)	15 (30.6)
**ZIKV Serostatus at baseline**			
Negative	86 (88.6)	47 (94)	39 (83)
Positive	11 (11.4)	3 (6)	8 (17)
**Yellow fever vaccine (Decade of Vaccination)**			
2020s	1 (1.01)	1 (2)	0
2010s	23 (23.3)	10 (20)	13 (26.5)
2000s	8 (8.06)	6 (12)	2 (4.08)
1990s	6 (6.03)	3 (6)	3 (6.12)
Unknown / No record	61 (61.6)	30 (60)	31 (63.3)
**Breadth of Response**			
No response	15 (15.8)	9 (18.7)	6 (12.8)
Narrow	17 (17.9)	14 (29.2)	3 (6.4)
Limited	16 (16.8)	8 (16.7)	8 (17.0)
Broad	35 (36.8)	13 (27.1)	22 (46.8)
Tetravalent	12 (12.6)	4 (8.3)	8 (17.0)

Suppl. Table 1
