# Supplementary material for: Serotype skewing and immune imprinting shape response to the tetravalent dengue virus Qdenga vaccine
Source: medRxiv. 2026 Jun 26:2026.06.15.26355542. Preprint. [Version 1] doi: 10.64898/2026.06.15.26355542 (PMC13321214; doi:10.64898/2026.06.15.26355542)
Supplement: Supplement 9 — Table S2. Reactogenicity profile following Qdenga vaccination. Summary of local and systemic symptoms observed or reported following the first and second doses of Qdenga. Immediate post-vaccination reactions were directly observed by clinical professionals during a 30-minute monitoring period, while subsequent events were self-reported by participants through structured questionnaires. Monitored symptoms included local reactions and systemic manifestations. Events were graded by intensity (mild, moderate, severe). Exact two-sided 95% confidence intervals were calculated using the Clopper–Pearson method for binomial proportions. The figure presents three panels: overall cohort (all participants), participants stratified by age group (<65 years, ≥65 years), and participants stratified by baseline DENV serostatus (DENV-naïve and DENV-exposed). [file media-11.pdf]

All Particpants<br>Event	After 1st dose<br>% (n, CI 95% Clopper-Pearson)	After 2st dose<br>% (n, CI 95% Clopper-Pearson)
**Local reactions** (at the application site)		
Pain	11.3% (11, 95% CI 5.8%–19.4%)	3.1% (3, 95% CI 0.6%–8.8%)
Hematoma/Ecchymosis/Hypersensitivity	1.0% (1, 95% CI 0.0%–5.6%)	3.1% (3, 95% CI 0.6%–8.8%)
Erythema/Redness	–	2.1% (2, 95% CI 0.3%–7.3%)
**Systemic**		
Fever	3.1% (3, 95% CI 0.6%–8.8%)	2.1% (2, 95% CI 0.3%–7.3%)
Nausea	3.1% (3, 95% CI 0.6%–8.8%)	-
Flu-like ilness	2.1% (2, 95% CI 0.3%–7.3%)	3.1% (3, 95% CI 0.6%–8.8%)
Diarrhea	2.1% (2, 95% CI 0.3%–7.3%)	–
Malaise/Fatigue/Myalgia	1.0% (1, 95% CI 0.0%–5.6%)	3.1% (3, 95% CI 0.6%–8.8%)
Headache	–	2.1% (2, 95% CI 0.3%–7.3%)
Retro-orbital pain	1.0% (1, 95% CI 0.0%–5.6%)	1.0% (1, 95% CI 0.0%–5.6%)
Chest pain	1.0% (1, 95% CI 0.0%–5.6%)	–
Acute coronary syndrome	–	1.0% (1, 95% CI 0.0%–5.6%)
**Any Local**	12.4% (12, 95% CI 6.6%–20.6%)	8.2% (8, 95% CI 3.6%–15.6%)
**Any Systemic**	13.4% (13, 95% CI 7.3%–21.8%)	12.4% (12, 95% CI 6.6%–20.6%)
**<7 Days**	21.6% (21, 95% CI 13.9%–31.2%)	18.6% (18, 95% CI 11.4%–27.7%)
**8-30 Days**	4.1% (4, 95% CI 1.1%–10.2%)	2.1% (2, 95% CI 0.3%–7.3%)

18-64 years old<br>Event	After 1st dose<br>% (n, CI 95% Clopper-Pearson)	After 2st dose<br>% (n, CI 95% Clopper-Pearson)
**Local reactions** (at the application site)		
Pain	20.4% (10, 95% CI 10.2%–34.3%)	6.1% (3, 95% CI 1.3%–16.9%)
Hematoma/Ecchymosis/Hypersensitivity	2.0% (1, 95% CI 0.1%–10.9%)	-
Erythema/Redness	-	2.0% (1, 95% CI 0.1%–10.9%)
**Systemic**		
Fever	6.1% (3, 95% CI 1.3%–16.9%)	2.0% (1, 95% CI 0.1%–10.9%)
Nausea	-	-
Flu-like ilness	4.1% (2, 95% CI 0.5%–14.0%)	2.0% (1, 95% CI 0.1%–10.9%)
Diarrhea	4.1% (2, 95% CI 0.5%–14.0%)	-
Malaise/Fatigue/Myalgia	6.1% (3, 95% CI 1.3%–16.9%)	4.1% (2, 95% CI 0.5%–14.0%)
Headache	-	2.0% (1, 95% CI 0.1%–10.9%)
Retro-orbital pain	-	2.0% (1, 95% CI 0.1%–10.9%)
Chest pain	-	-
Acute coronary syndrome	-	-
**Any Local**	22.4% (11, 95% CI 11.8%–36.6%)	8.2% (4, 95% CI 2.3%–19.6%)
**Any Systemic**	20.4% (10, 95% CI 10.2%–34.3%)	12.2% (6, 95% CI 4.6%–24.8%)
**<7 Days**	24.5% (12, 95% CI 13.3%–38.9%)	20.4% (10, 95% CI 10.2%–34.3%)
**8-30 Days**	4.1% (2, 95% CI 0.5%–14.0%)	0.0% (0, 95% CI 0.0%–7.3%)

65+ years old<br>Event	After 1st dose<br>% (n, CI 95% Clopper-Pearson)	After 2st dose<br>% (n, CI 95% Clopper-Pearson)
**Local reactions** (at the application site)		
Pain	2.1% (1, 95% CI 0.1%–11.1%)	-
Hematoma/Ecchymosis/Hypersensitivity	-	4.2% (2, 95% CI 0.5%–14.3%)
Erythema/Redness	-	4.2% (2, 95% CI 0.5%–14.3%)
**Systemic**		
Fever	-	2.1% (1, 95% CI 0.1%–11.1%)
Nausea	2.1% (1, 95% CI 0.1%–11.1%)	-
Flu-like ilness	-	4.2% (2, 95% CI 0.5%–14.3%)
Diarrhea	-	-
Malaise/Fatigue/Myalgia	-	2.1% (1, 95% CI 0.1%–11.1%)
Headache	-	2.1% (1, 95% CI 0.1%–11.1%)
Retro-orbital pain	2.1% (1, 95% CI 0.1%–11.1%)	-
Chest pain	2.1% (1, 95% CI 0.1%–11.1%)	-
Acute coronary syndrome	-	2.1% (1, 95% CI 0.1%–11.1%)
**Any Local**	2.1% (1, 95% CI 0.1%–11.1%)	8.3% (4, 95% CI 2.3%–20.0%)
**Any Systemic**	6.3% (3, 95% CI 1.3%–17.2%)	20.8% (10, 95% CI 10.5%–35.0%)
**<7 Days**	4.2% (2, 95% CI 0.5%–14.3%)	16.7% (8, 95% CI 7.5%–30.2%)
**8-30 Days**	4.2% (2, 95% CI 0.5%–14.3%)	4.2% (2, 95% CI 0.5%–14.3%)

DENV-naïve<br>Event	After 1st dose<br>% (n, CI 95% Clopper-Pearson)	After 2st dose<br>% (n, CI 95% Clopper-Pearson)
**Local reactions** (at the application site)		
Pain	16.3% (8, 95% CI 7.3%–29.7%)	2.0% (1, 95% CI 0.1%–10.9%)
Hematoma/Ecchymosis/Hypersensitivity	-	2.0% (1, 95% CI 0.1%–10.9%)
Erythema/Redness	-	2.0% (1, 95% CI 0.1%–10.9%)
**Systemic**		
Fever	4.1% (2, 95% CI 0.5%–14.0%)	4.1% (2, 95% CI 0.5%–14.0%)
Nausea	4.1% (2, 95% CI 0.5%–14.0%)	-
Flu-like ilness	2.0% (1, 95% CI 0.1%–10.9%)	4.1% (2, 95% CI 0.5%–14.0%)
Diarrhea	-	-
Malaise/Fatigue/Myalgia	2.0% (1, 95% CI 0.1%–10.9%)	2.0% (1, 95% CI 0.1%–10.9%)
Headache	-	2.0% (1, 95% CI 0.1%–10.9%)
Retro-orbital pain	-	-
Chest pain	-	-
Acute coronary syndrome	-	-
**Any Local**	16.3% (8, 95% CI 7.3%–29.7%)	6.1% (3, 95% CI 1.3%–16.9%)
**Any Systemic**	12.2% (6, 95% CI 4.6%–24.8%)	18.4% (9, 95% CI 8.8%–32.0%)
**<7 Days**	24.5% (12, 95% CI 13.3%–38.9%)	18.4% (9, 95% CI 8.8%–32.0%)
**8-30 Days**	6.1% (3, 95% CI 1.3%–16.9%)	0.0% (0, 95% CI 0.0%–7.3%)

DENV-exposed<br>Event	After 1st dose<br>% (n, CI 95% Clopper-Pearson)	After 2st dose<br>% (n, CI 95% Clopper-Pearson)
**Local reactions** (at the application site)		
Pain	6.3% (3, 95% CI 1.3%–17.2%)	4.2% (2, 95% CI 0.5%–14.3%)
Hematoma/Ecchymosis/Hypersensitivity	2.1% (1, 95% CI 0.1%–11.1%)	2.1% (1, 95% CI 0.1%–11.1%)
Erythema/Redness	-	4.2% (2, 95% CI 0.5%–14.3%)
**Systemic**		
Fever	2.1% (1, 95% CI 0.1%–11.1%)	-
Nausea	2.1% (1, 95% CI 0.1%–11.1%)	-
Flu-like ilness	2.1% (1, 95% CI 0.1%–11.1%)	2.1% (1, 95% CI 0.1%–11.1%)
Diarrhea	4.2% (2, 95% CI 0.5%–14.3%)	-
Malaise/Fatigue/Myalgia	-	4.2% (2, 95% CI 0.5%–14.3%)
Headache	-	2.1% (1, 95% CI 0.1%–11.1%)
Retro-orbital pain	-	2.1% (1, 95% CI 0.1%–11.1%)
Chest pain	2.1% (1, 95% CI 0.1%–11.1%)	-
Acute coronary syndrome	-	2.1% (1, 95% CI 0.1%–11.1%)
**Any Local**	8.3% (4, 95% CI 2.3%–20.0%)	10.4% (5, 95% CI 3.5%–22.7%)
**Any Systemic**	12.5% (6, 95% CI 4.7%–25.2%)	12.5% (6, 95% CI 4.7%–25.2%)
**<7 Days**	18.8% (9, 95% CI 8.9%–32.6%)	18.8% (9, 95% CI 8.9%–32.6%)
**8-30 Days**	2.1% (1, 95% CI 0.1%–11.1%)	4.2% (2, 95% CI 0.5%–14.3%)

Suppl. Table 2
