# Supplementary material for: Serotype skewing and immune imprinting shape response to the tetravalent dengue virus Qdenga vaccine
Source: medRxiv. 2026 Jun 26:2026.06.15.26355542. Preprint. [Version 1] doi: 10.64898/2026.06.15.26355542 (PMC13321214; doi:10.64898/2026.06.15.26355542)
Supplement: Supplement 10 — Table S3. Geometric mean neutralizing antibody titers (GMTs) against DENV-1–4 vaccinal strains following Qdenga vaccination. Neutralization was measured by focus reduction neutralization test (FRNT) using plasma collected at baseline (T0), at the time of the second vaccination dose (90 days after the first dose, T1), and at 7 days (T2) and 60 days (T3) post-second dose. Participants were stratified by baseline dengue serostatus (DENV-naïve and DENV-exposed). Data are shown as GMTs with 95% confidence intervals (CIs) for each DENV serotype. [file media-12.pdf]

Geometric Mean Titer - 95% CI

	DENV-naive	DENV-exposed
**DENV 1**		
T0	10.086 (10.035–10.138)	95.147 (58.773-154.034)
T1	40.037 (26.672–60.099)	752.365 (431.049-1313.199)
T2	43.758 (28.913–66.225)	936.483 (556.819-1575.019)
T3	47.565 (30.098–75.171)	1094.213 (629.452-1902.134)
**DENV 2**		
T0	10.835 (10.115–11.607)	24.417 (17.569-33.933)
T1	52.820 (37.876–73.660)	180.751 (125.778-259.752)
T2	63.301 (44.390–90.267)	181.621 (128.661-256.381)
T3	81.517 (54.538–121.843)	170.953 (118.204-247.242)
**DENV 3**		
T0	11.540 (9.389–14.186)	25.828 (18.306-36.440)
T1	41.580 (30.415–56.843)	194.911 (116.765-325.358)
T2	27.966 (21.123–37.026)	141.933 (85.287-236.201)
T3	32.107 (23.902–43.129)	232.667 (151.340-357.698)
**DENV 4**		
T0	10.056 (9.997–10.115)	10.868 (10.195-11.585)
T1	11.619 (10.258–13.160)	19.225 (15.367-24.053)
T2	11.449 (10.133–12.936)	18.885 (14.970-23.824)
T3	11.764 (10.510–13.168)	17.830 (14.332-22.184)

Suppl. Table 3
