# Supplementary material for: Serotype skewing and immune imprinting shape response to the tetravalent dengue virus Qdenga vaccine
Source: medRxiv. 2026 Jun 26:2026.06.15.26355542. Preprint. [Version 1] doi: 10.64898/2026.06.15.26355542 (PMC13321214; doi:10.64898/2026.06.15.26355542)
Supplement: Supplement 2 — Supplementary Data Figure 2. Characterization of B cell subpopulations dynamics following Qdenga vaccination. Frequencies and gating strategies for major B cell subsets gated on live, single CD19+CD20+ cells. Samples were collected at four time points: T0 (baseline, prior to first dose), T1 (90 days post-first dose/before the second dose), T2 (7 days post-second dose), and T3 (60 days post-second dose). Participants were stratified by baseline serostatus: DENV-naïve (blue, n = 47) and DENV-exposed (purple, n = 45). Frequencies and representative plots of (a,b) total B cells, (c,d) early differentiated B cells (CD27−CD180+), (e,f) inflammatory B cells (CXCR3+HLA-DR+), (g,h) activated B cells (HLA-DR+CD86+), and (I,j) naïve B cells (CD27−CD38−) across all participants (red, left) and stratified by baseline serostatus: DENV-naïve (blue) and DENV-exposed (purple). Percentages for each gated population are indicated on the plots. Antibody-secreting cells, ASCs. Memory B cells, MBCs. Significance was determined using the Kruskal–Wallis test followed by Dunn’s multiple comparisons test. Flow-cytometry gating was used to identify and quantify distinct B cell populations within peripheral blood mononuclear cells. Each dot in the plots represents an individual cell; the clusters enclosed by gates (boxes) correspond to the subpopulation of interest, and the percentage displayed indicates its frequency within the parent population. ****P < 0.0001, ***P < 0.001, **P < 0.01, and *P < 0.05. [file media-2.pdf]

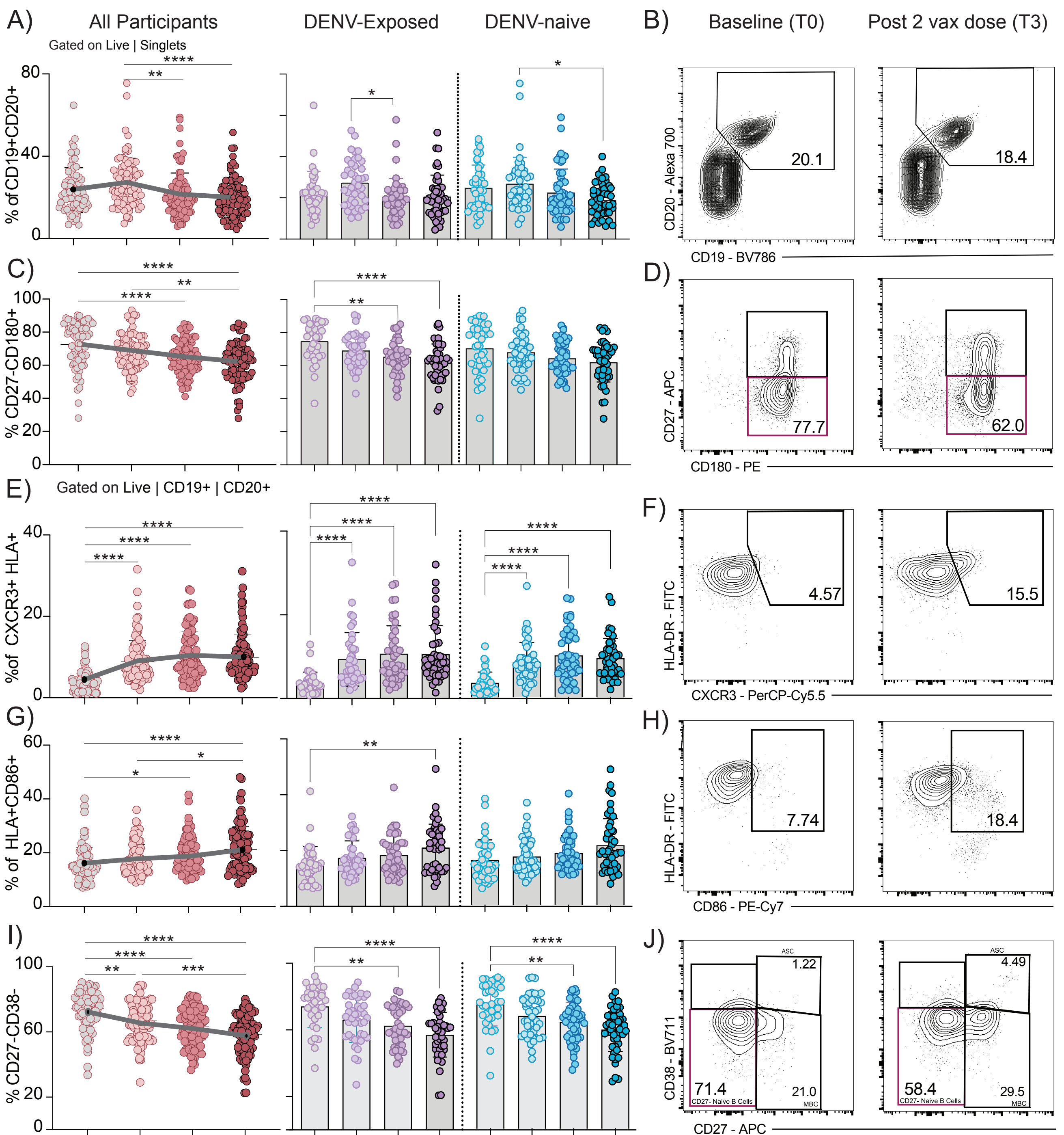
A)
All Participants
DENV-Exposed
DENV-naive
Gated on Live | Singlets
% of CD19+CD20+
B)
Baseline (T0)
Post 2 vax dose (T3)
CD20 - Alexa 700
CD19 - BV786
20.1
18.4
C)
% CD27-CD180+
D)
CD27 - APC
CD180 - PE
77.7
62.0
E)
Gated on Live | CD19+ | CD20+
%of CXCR3+ HLA+
F)
HLA-DR - FITC
CXCR3 - PerCP-Cy5.5
4.57
15.5
G)
% of HLA+CD86+
H)
HLA-DR - FITC
CD86 - PE-Cy7
7.74
18.4
I)
% CD27-CD38-
J)
CD38 - BV711
CD27 - APC
ASC
1.22
4.49
71.4
58.4
CD27- Naive B Cells
21.0
29.5
MBC
T0
T1
T2
T3

Suppl. Fig. 2
