# Supplementary material for: Serotype skewing and immune imprinting shape response to the tetravalent dengue virus Qdenga vaccine
Source: medRxiv. 2026 Jun 26:2026.06.15.26355542. Preprint. [Version 1] doi: 10.64898/2026.06.15.26355542 (PMC13321214; doi:10.64898/2026.06.15.26355542)
Supplement: Supplement 3 — Supplementary Data Figure 3. Characterization of T cell subpopulations induced by Qdenga vaccination. a–b. Frequencies of CD4+ (a) and CD8+ (b) T cell subsets defined by CCR7 and CD45RA expression: naïve (CCR7+CD45RA+), central memory (TCM, CCR7+CD45RA−), effector memory (TEM, CCR7−CD45RA−), and terminally differentiated effector memory (TEMRA, CCR7−CD45RA+). Data are shown at baseline (T0, prior to first dose, n = 77), 7 days post-second dose (T2, n = 92), and 60 days post-second dose (T3, n = 80). c–d. Frequency of CD4+PD1+TIM3+ (c) and CD8+PD1+TIM3+ (d) T cells at T0 and T3, stratified by baseline serostatus: DENV-naïve (blue, T0: n = 40; T2: n = 48; T3: n. = 40) and DENV-exposed (purple, T0: n = 37; T2: n = 44; T3: n. = 40). Significance was determined using the Kruskal–Wallis test followed by Dunn’s multiple comparisons test. e–f, Principal component analysis (PCA) of total T cell subsets showing no clustering by (e) age or (f) sex at T3. g–h. DENV-reactive T cell responses following stimulation with PrM and E peptide pools. Frequencies and distribution of AIM+ T cell subpopulations at T0 and T3: (g) AIM+ CD4+ T cells (OX40+CD137+) and (h) AIM+ CD8+ T cells (CD69+CD137+) further stratified by CCR7 and CD45RA expression into naïve, TCM, TEM, and TEMRA subsets stratified by baseline serostatus: DENV-naïve (blue, T0: n = 20; T3: n. = 41) and DENV-exposed (purple, T0: n = 22; T3: n. = 39). Statistical significance was assessed using a two-tailed Mann–Whitney test. Data are stratified by baseline serostatus: DENV-exposed (purple, V0, n = 49 V2, n = 49 V3, n = 49) and DENV-naïve (blue, n = 50) groups. Flow-cytometry gating was used to identify and quantify distinct T cell populations within peripheral blood mononuclear cells. Each dot in the plots represents an individual cell; the clusters enclosed by gates (boxes) correspond to the subpopulation of interest, and the percentage displayed indicates its frequency within the parent population.****P < 0.0001, ***P < 0.0 [file media-3.pdf]

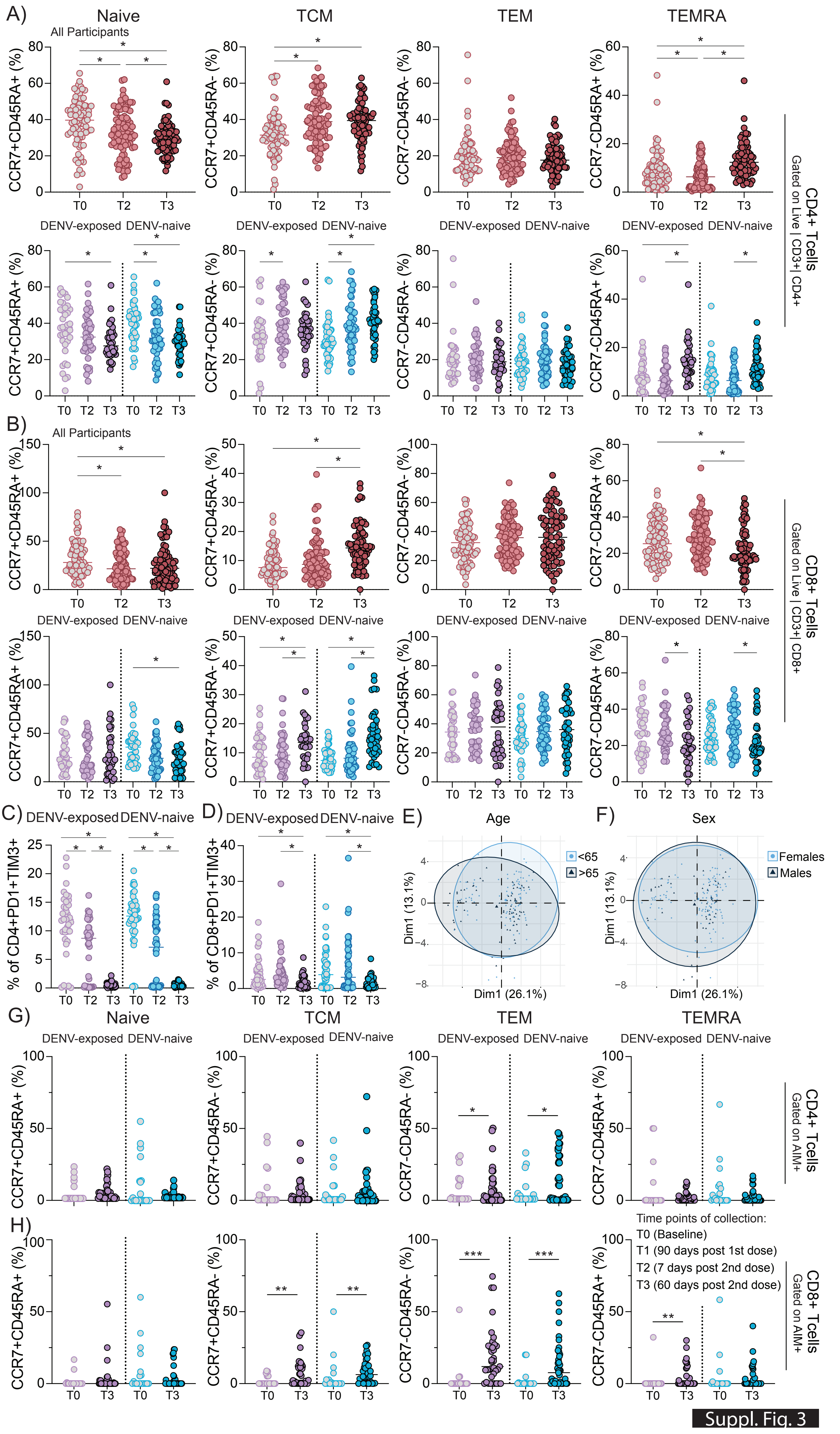
A)
Naive
TCM
TEM
TEMRA
All Participants
CCR7+CD45RA+ (%)
CCR7+CD45RA- (%)
CCR7-CD45RA- (%)
CCR7-CD45RA+ (%)
T0
T2
T3
DENV-exposed DENV-naive
CD4+ Tcells
Gated on Live | CD3+| CD4+
B)
CD8+ Tcells
Gated on Live | CD3+| CD8+
C)
DENV-exposed DENV-naive
% of CD4+PD1+TIM3+
D)
% of CD8+PD1+TIM3+
E)
Age
<65
>65
Dim1 (13.1%)
Dim1 (26.1%)
F)
Sex
Females
Males
G)
CD4+ Tcells
Gated on AIM+
H)
CD8+ Tcells
Gated on AIM+
Time points of collection:
T0 (Baseline)
T1 (90 days post 1st dose)
T2 (7 days post 2nd dose)
T3 (60 days post 2nd dose)
Suppl. Fig. 3
